# Serendipitous Discovery of T Cell–Produced KLK1b22 as a Regulator of Systemic Metabolism

**DOI:** 10.4049/immunohorizons.2300016

**Published:** 2023-06-26

**Authors:** Matthew L. Arwood, Im-Hong Sun, Chirag H. Patel, Im-Meng Sun, Min-Hee Oh, Ian A. Bettencourt, Michael D. Claiborne, Yee Chan-Li, Liang Zhao, Adam T. Waickman, Orestes Mavrothalassitis, Jiayu Wen, Susan Aja, Jonathan D. Powell

**Affiliations:** * Bloomberg-Kimmel Institute for Cancer Immunotherapy, Sidney-Kimmel Comprehensive Cancer Research Center, Johns Hopkins University School of Medicine, Baltimore, MD; † Department of Medicine, Johns Hopkins University School of Medicine, Baltimore, MD; ‡ State University of New York Upstate Medical University, Syracuse, NY; § Department of Anesthesia, University of California, San Francisco School of Medicine, San Francisco, CA; ¶ Center for Metabolism and Obesity Research, Johns Hopkins Medicine, Baltimore, MD; ‖ Solomon H. Snyder Department of Neuroscience, Johns Hopkins University School of Medicine, Baltimore, MD

## Abstract

In order to study mechanistic/mammalian target of rapamycin’s role in T cell differentiation, we generated mice in which Rheb is selectively deleted in T cells (T-Rheb^−/−^ C57BL/6J background). During these studies, we noted that T-Rheb^−/−^ mice were consistently heavier but had improved glucose tolerance and insulin sensitivity as well as a marked increase in beige fat. Microarray analysis of Rheb^−/−^ T cells revealed a marked increase in expression of kallikrein 1–related peptidase b22 (*Klk1b22*). Overexpression of KLK1b22 in vitro enhanced insulin receptor signaling, and systemic overexpression of KLK1b22 in C57BL/6J mice also enhances glucose tolerance. Although KLK1B22 expression was markedly elevated in the T-Rheb^−/−^ T cells, we never observed any expression in wild-type T cells. Interestingly, in querying the mouse Immunologic Genome Project, we found that *Klk1b22* expression was also increased in wild-type 129S1/SVLMJ and C3HEJ mice. Indeed, both strains of mice demonstrate exceptionally improved glucose tolerance. This prompted us to employ CRISPR-mediated knockout of KLK1b22 in 129S1/SVLMJ mice, which in fact led to reduced glucose tolerance. Overall, our studies reveal (to our knowledge) a novel role for KLK1b22 in regulating systemic metabolism and demonstrate the ability of T cell–derived KLK1b22 to regulate systemic metabolism. Notably, however, further studies have revealed that this is a serendipitous finding unrelated to Rheb.

## Introduction

The mechanistic/mammalian target of rapamycin (mTOR) pathway has emerged as a critical regulator of innate and adaptive immune responses (1, 2). Our group and others have shown that mTOR complex 1 (mTORC1) and mTOR complex 2 (mTORC2) are key nodes in regulating both CD4^+^ and CD8^+^ T cell differentiation (3–9). In CD4^+^ cells, mTORC1 supports the differentiation of Th1 and Th17 cells, whereas mTORC2 supports Th2 cells. Furthermore, inhibiting mTOR kinase signaling promotes T regulatory cell differentiation. In CD8^+^ cells, mTORC1 promotes the development of effector cells, whereas mTORC2 inhibits the generation of memory T cells. Proper mTORC1 activation is promoted by the upstream molecule Ras homolog enriched in brain (Rheb) (1). Rheb is a small guanosine triphosphatase localized to the lysosome where the mTORC1 complex is activated.

Kallikreins (KLKs) are a diverse class of proteases. In humans, KLKs are divided into two groups: plasma KLK (KLK1b), which is produced in the liver and circulates in the blood; and tissue KLKs, which are produced in various tissues throughout the body (10, 11). It has been shown that plasma KLK is important in cardiovascular regulation (12, 13). Furthermore, plasma KLK has a role in the development of diabetes, and thus several groups have developed plasma KLK inhibitors (12, 13). The tissue KLK family consists of 15 serine proteases and is the largest protease gene grouping in the human genome (10). A well-studied tissue KLK is KLK3, also known as prostate-specific Ag. Prostate-specific Ag levels are increased in prostate cancer and are used to screen for the disease (14). There is also evidence for human KLK1 in mediating blood glucose, which has led to further preclinical studies (15–17). Interestingly, in mice, a duplication of the *Klk1* gene led to 13 additional genes called the “kallikrein 1-related peptidases family b” (18–20).

While performing immunological studies with conditional floxed Rheb-knockout (KO) mice (T-Rheb^−/−^), which have Rheb deleted specifically in CD4^+^ and CD8^+^ cells through *Cd4*-driven Cre recombinase expression, we observed that the T-Rheb^−/−^ mice were heavier than the wild-type (WT) control mice (5). Because these mice had increased weight and adipose tissue, it was originally thought that the T-Rheb^−/−^ mice might have an increased risk of developing insulin-resistant diabetes. In fact, this hypothesis was completely wrong. It turns out that these heavier T-Rheb^−/−^ mice were found actually to have an improved metabolic state, with lower fasting blood glucose than their WT counterparts, even when challenged with a high-fat diet (HFD). Microarray analysis of the Rheb^−/−^ T cells revealed, to our knowledge, a novel expression of *kallikrein 1-related peptidase b22* (*Klk1b22*). KLK1b22 is a trypsin-like serine protease that is normally expressed in the mouse salivary gland, where its role was originally described to bind epidermal and/or nerve growth factors in the saliva (21–25). In this study, we report that T cell–derived KLK1b22 can reduce fasting blood glucose and improve systemic glucose tolerance.

## Materials and Methods

### Mice

Male and female mice aged 1.5 to 6 mo old were used for all the experiments in this study. Mice were age and sex matched for each experiment. C57BL/6J, *Cd4*-Cre, 129S1/SVLMJ, C3HEJ, and *Rag2*^−/−^ mice were obtained from The Jackson Laboratory. WT control mice for each experiment were C57BL/6J mice obtained from The Jackson Laboratory that were bred in-house. Mice with loxP-flanked *Rheb* alleles were gifted by Dr. Paul Worley (Johns Hopkins University School of Medicine). These loxP-flanked *Rheb* mice were then bred to *Cd4-Cre* mice to obtain a T cell–specific Rheb KO (5). KLK1b22 CRISPR KO mice were generated on a 129S1/SVLMJ background in cooperation with the Transgenic Core Laboratory at the Johns Hopkins University School of Medicine. Guide RNAs were designed using the Breaking-Cas online design tool (26). Each injection consisted of a mixture of two different guide RNAs (5′-CTTAGATGAGTACCTATGCG-3′ and 5′-TCCTCCAAAGTGTACCTACT-3′). CRISPR KO mice were genotyped via PCR with the following primers (forward: 5′-GCTCCCTTTCCTGGATTCATTTAT-3′, reverse: 5′-TCACACAGACCTCATTAGGATGGA-3′). For diet-induced obesity, mice that were 6 wk old were fed ad libitum with an OpenSource diet containing 60% kcal fat (Research Diets D12492).

All mouse procedures were approved by the Johns Hopkins University Institutional Animal Care and Use Committee (M019M71). Furthermore, all mouse procedures and methods were carried out and reported in accordance with Animal Research: Reporting of In Vivo Experiments and institutional guidelines. All methods were carried out in accordance with relevant guidelines and regulations. All methods, husbandry procedures, housing conditions, and animal handling procedures were performed in line with the Guide for the Care and Use of Laboratory Animals (Institute for Laboratory Animal Research of the National Research Council of the National Academies).

### Body fat composition and serum chemistry (lipid panel)

Adipose weight, body fat percentage, lean weight and body lean percentage were determined and calculated by QMR (EchoMRI-100).

Mice were fasted overnight, and blood was collected via cardiac puncture. Blood was allowed to clot at room temperature, then centrifuged, and serum was isolated and stored at −80°C. Serum was analyzed for total cholesterol, high-density lipoprotein (HDL), and triglycerides on an Alfa Wasserman VetACE chemistry analyzer. These machines are maintained by the Phenotyping Core in the Department of Molecular and Comparative Pathobiology at the Johns Hopkins University School of Medicine.

### ELISA

Mice were fasted overnight, and blood was collected via cardiac puncture. Blood was allowed to clot at room temperature, then centrifuged, and serum was isolated and frozen at −80°C. Serum was analyzed via ELISA for rat/mouse insulin (Millipore EZRMI-13K), mouse adiponectin (Millipore EZMADP-60K), and mouse leptin (Millipore EZML-82K) per the manufacturer’s instructions.

### Blood glucose measurements

Procedures described below were adapted from Andrikopoulos et al. (27) and Ayala et al. (28). All blood glucose measurements were obtained using a One Touch Ultra 2 blood glucose meter. Blood was obtained via clipping the distal 1–2 mm of tail.

For i.p. glucose tolerance tests (IP-GTTs), after 6–8 h of fasting, a fasting blood glucose measurement was obtained. Then a solution of sterile filtered d-glucose in water was injected i.p. with a final amount of glucose of 1 g, 1.5 g, or 2 g glucose per 1 kg body weight. Blood glucose readings were then collected serially.

For i.p. insulin tolerance tests, after 2 h of fasting, a baseline blood glucose measurement was obtained. Then a solution of insulin in PBS was injected i.p. with a final amount of insulin of 0.5 U or 1 U per 1 kg body weight. Blood glucose readings were then collected serially.

### Real-time PCR (quantitative RT-PCR)

Total RNA from cells was extracted with TRIzol reagent. For RNA analysis from blood, mice were cheek bled into a mixture of sodium citrate (final 2%) and RPMI. The cell pellet was incubated with ammonium-chloride-potassium (ACK) lysis buffer. The remaining pellet was suspended in TRIzol for RNA extraction. Total RNA from tissue was isolated by homogenization with an Omni Tissue Homogenizer in QIAzol Lysis Reagent using an RNeasy Lipid Tissue Mini Kit (Qiagen 74804). RNA was converted to cDNA using ProtoScript II Reverse Transcriptase. Predesigned TaqMan gene expression assays were purchased from Applied Biosystems: *Klk1b22* (Mm02343755_g1, Mm02343756_g1, Mm02343754_m1), uncoupling protein 1 (*Ucp1*) (Mm01244861_m1), *Cidea* (Mm00432554_m1), *Cpt1b* (Mm01308160_m1), *Prdm16* (Mm00712556_m1), *Tbx21* (Mm00450960_m1), *Gata3* (Mm00484683_m1), *Rorc* (Mm01261022_m1), *Ifng* (Mm01168134_m1), and *Ppargc1a* (*Pgc1*α) (Mm01208835_m1). Quantitative RT-PCR (qRT-PCR) was performed with EagleTaq Universal Master Mix using an Applied Biosystems StepOnePlus 96-well RT-PCR. ΔΔCt values were normalized to 18S rRNA (Applied Biosystems 4310893E) and to a control group of interest or background.

### Western blotting

Cells were directly lysed in radioimmunoprecipitation assay buffer (Abcam Western blotting protocol) containing cOmplete protease inhibitor mixture (Roche 11697498001), sodium fluoride, PMSF, sodium pyrophosphate, β-glycerophosphate, and sodium orthovanadate. Tissue was homogenized with an Omni Tissue Homogenizer in radioimmunoprecipitation assay buffer containing the above additives. Protein concentration was determined via the Coomassie protein assay reagent. Western blotting was performed using a standard protocol (Abcam Western blotting protocol). When using serum for Western blotting, polyvinylidene difluoride membranes were incubated with Ponceau solution prior to blocking to stain for total protein. After washing with TBST, membranes were blocked with milk solution, and Western blotting continued.

The following Abs were purchased from Cell Signaling Technology: anti-succinate dehydrogenase (SDHA) (D6J9M, 11998), anti-voltage-dependent anion channel (VDAC) (D73D12, 4661), anti-phospho-IGF-I receptor β (Tyr1131)/insulin receptor β (Tyr1146) (3021), anti-insulin receptor β (4B8, 3025), glucose transporter 4 (Glut4) (1F8, 2213), and anti-β-actin (D6A8, 8457). The following Abs were purchased from Abcam: anti-UCP-1 (ab10983), and anti-PGC1α (ab54481). The following Ab was purchased from Sigma-Aldrich: anti-actin (A2066). The following Ab was produced by Creative Biolabs as a custom rat hybridoma mAb production project: KLK1b22 (14C5). The proteins were detected with ECL Plus substrate or SuperSignal West Pico PLUS chemiluminescent substrate. The images were captured using a UVP BioSpectrum 500 Imaging System and analyzed with the accompanying software.

### Adipose tissue explant

This explant protocol was adapted from Pedersen et al. (29). Recombinant KLK1b22 was activated by thermolysin as outlined in the *Recombinant KLK1b22* section. Inguinal adipose tissue was harvested from WT C57BL/6J mice, cut into small pieces, and distributed into culture media (DMEM supplemented with 0.5 mM glucose, 2 mM sodium pyruvate, 2 mM glutamine, and antibiotics). Adipose tissue explants were incubated in the presence or absence of thermolysin-activated KLK for 1.5 h at 37°C and were subsequently stimulated in the presence or absence of 100 nM insulin for 45 min at 37°C. Tissue explants were washed with ice-cold PBS once and then snap frozen. The tissue was then processed for Western blotting as outlined in the *Western Blotting* section.

### Immunohistochemistry

Protocols were followed as outlined by Chi and Chandy (30) and Qiu et al. (31). Mice were sacrificed and perfused with PBS. Then inguinal white adipose tissue (WAT) was dissected out and fixed in 10% formalin overnight. Samples were then washed and stored in 70% ethanol until being embedded in paraffin. The tissue was cut in 4-mm sections and mounted on slides. Slides were stained with anti-UCP-1 (Abcam ab10983) and then developed with the VECTASTAIN ABC-HRP Kit (Vector Laboratories PK-4000) and DAB Peroxidase Substrate (Vector Laboratories SK4100). Samples were counterstained with hematoxylin.

### Flow cytometry

Procedures for adipose tissue flow cytometry were adapted from Orr et al. (32). Perigonadal WAT was dissected out of mice and digested in 2 mg/ml collagenase type II for 20 min at 37°C. The reaction was quenched with EDTA (15 mM) and ice incubation. The sample was then filtered through a 70-μm cell strainer and incubated with ACK lysis buffer. Spleens were dissected out of mice, mashed through a 70-μm cell strainer, and incubated with ACK lysis buffer.

The following Abs were purchased from BioLegend: BV510 anti-CD45 (30-F11, 103138), PE anti-F4/80 (BM8, 123110), Alexa Fluor 700 anti-CD11b (M1/70, 101222), BV650 anti-CD206 (C068C2, 141723), BV605 anti-CD8a (53-6.7, 100744), BV785 anti-CD25 (PC61, 102051), FITC anti-CD19 (6D5, 115506), and PerCP/cyanine 5.5 anti-CD3ε (145-2C11, 100328). The following Abs were purchased from BD Biosciences: anti-CD16/CD32 (mouse BD Fc block) (2.4G2, 553142), FITC anti-CD11c (HL3, 557400), allophycocyanin anti-Ly-6G and Ly-6C (RB6-8C5, 553129), FITC anti-CD8a (53-6.7, 553031), PE anti-CD4 (GK1.5, 553730), Alexa Fluor 647 anti-CD8a (53-6.7, 557682), allophycocyanin anti-CD4 (RM4-5, 553051), and PE-CF594 anti-CD4 (RM4-5, 562285). The following reagents and Abs were purchased from eBioscience: Fixable Viability Dye eFluor 780 (65-0865-14), FITC anti-CD3e (145-2C11, 11-0031-85), PerCP-cyanine 5.5 anti-Ly-6C (HK1.4, 45-5932-82), and eFluor 450 anti-FOXP3 (FJK-16s, 48-5773-82). The following reagent was purchased from Thermo Fisher Scientific: LIVE/DEAD Fixable Near-IR Dead Cell Stain Kit (L34976). The following Ab was purchased from Bio X Cell: InVivoMAb anti-CD16/CD32 (2.4G2, BE0307).

For intracellular staining, the eBioscience fixation/permeabilization kit was used (88-8824-00). After staining, all experiments were run on a BD FACSCalibur or BD FACSCelesta. Data were analyzed with FlowJo analysis software. Some samples were stained and sorted using a BD FACSAria II cytometer. These samples were from spleen and bone marrow. From spleen, T cells (CD3^+^) and B cells (CD19^+^) were obtained. From bone marrow, neutrophils (CD11b^+^, Ly6G^+^) and monocytes (CD11b^+^, Ly6C^+^) were obtained. After sorting, these samples were pelleted, then lysed in TRIzol, and qRT-PCR was performed as described in the *Real-Time PCR (Quantitative RT-PCR)* section above.

### Microarray

CD4^+^ and CD8^+^ T cells were isolated by MACS cell separation (Miltenyi Biotec) from spleens of WT and T-Rheb^−/−^ mice. Single biological samples were submitted to the Johns Hopkins Deep Sequencing and Microarray Core Facility and run on an Affymetrix Mouse Exon 1.0 ST Array. These data have been deposited to the Gene Expression Omnibus databank (https://www.ncbi.nlm.nih.gov/geo/query/acc.cgi?acc=GSE156529).

### Adenovirus

Ad-GFP adenovirus (Ad) was purchased from Vector Biolabs. Ad-KLK1b22 adenovirus was generated using the Gateway Vector Kit adenoviral expression vector (Invitrogen) and amplified to a higher titer by Vector Biolabs. Adenovirus was stored in aliquots at −80°C until use. For in vivo use, adenovirus was diluted in PBS. WT mice were administered 2 × 10^9^ to 1 × 10^10^ PFU adenovirus via i.v. tail vein injection. This amount of active virus was similar to that used in previously published studies (33, 34). Fasting blood glucose was checked 2–3 wk after adenovirus injection, and IP-GTT was performed as indicated above in the *Blood Glucose Measurements* section.

### Recombinant KLK1b22

Recombinant KLK1b22 was generated by and purchased from Creative BioMart. The protein was produced in HEK293 cells. The supplied protein is pro-KLK1b22 and requires cleavage for full activation. For in vitro experiments, the activation protocol for recombinant human KLK1 (R&D Systems 2337-SE) was followed to cleave off the promoiety and fully activate the protein. In brief, recombinant KLK1b22 was incubated with 1 μg/ml thermolysin at 37°C for 1 h. This reaction was stopped by the addition of 1,10-phenanthroline to a final concentration of 10 mM.

For in vivo experiments, the supplied proprotein directly from Creative BioMart was used. WT mice were fasted for 7 h and then administered 43 μg recombinant KLK1b22 via i.v. tail vein injection. Blood glucose was measured 0.75 h after i.v. KLK1b22 administration. Then an IP-GTT was performed as outlined above in the *Blood Glucose Measurements* section.

### Phosphorylated insulin receptor stimulation and decay assays

HEK293T insulin receptor stimulation assays were adapted from Krishnan et al. (35). For adenovirus experiments, HEK293T were seeded onto poly-d-lysine–coated plates. The next day, cells were washed and incubated with virus at 25 multiplicities of infection for several hours, and then the virus was removed. The following day, cells were serum starved in 2% FBS-containing DMEM for 2 h. Then the cells were either stimulated with insulin (0.5 or 2 μg/ml) for the indicated durations or stimulated with insulin for 10 min, and then the insulin was washed out and the cells were rested in HBSS until harvesting for Western blot analysis.

For recombinant protein experiments, HEK293T cells were seeded onto poly-d-lysine–coated plates. The following day, cells were serum starved in 2% FBS-containing DMEM for 2 h. For the last 30 min of serum starvation, cells were incubated in 5 μg/ml of thermolysin-activated recombinant KLK1b22 (protocol outlined in the *Recombinant KLK1b22* section above). At the end of serum starvation, the cells were stimulated with insulin (0.5 μg/ml) for 10 min, and then the insulin was washed out and the cells were rested in HBSS until harvesting for Western blot analysis.

### Adoptive transfer

Adoptive transfer experiments were performed by transferring cells via retro-orbital injection into *Rag2*^−/−^ mice. In brief, spleens and lymph nodes of WT C57BL/6J or 129S1/SvImJ mice were harvested and mashed through a 70-μm cell strainer. Then cells were isolated with a mouse CD8a^+^ T Cell Isolation Kit (Miltenyi Biotec 130-104-075). Purity was then checked via flow cytometry. A total of 1 × 10^6^ CD8^+^ cells were then transferred into *Rag2*^−/−^ mice. Cells were allowed to homeostatically proliferate. Mice were cheek bled periodically to check engraftment of the cells. Ten to 24 d after cell transfer, mice were fasted, and an IP-GTT was performed.

### Statistical analysis

All of the graphs were created and statistical analyses performed using GraphPad Prism software. The specific statistical tests performed are the recommended tests from GraphPad Prism and are outlined in each figure legend. A D’Agostino and Pearson normality test or a Shapiro-Wilk normality test was performed to determine if the data had a normal Gaussian distribution and guide in the use of a parametric versus nonparametric test. A *p* value less than 0.05 was considered statistically significant (*p ≤ 0.05, **p ≤ 0.01, ***p ≤ 0.001, ****p ≤ 0.0001). The SEM is shown for all data.

## Results

### T-Rheb^−/−^ mice have improved glucose use despite increased weight

**FIGURE 1.**
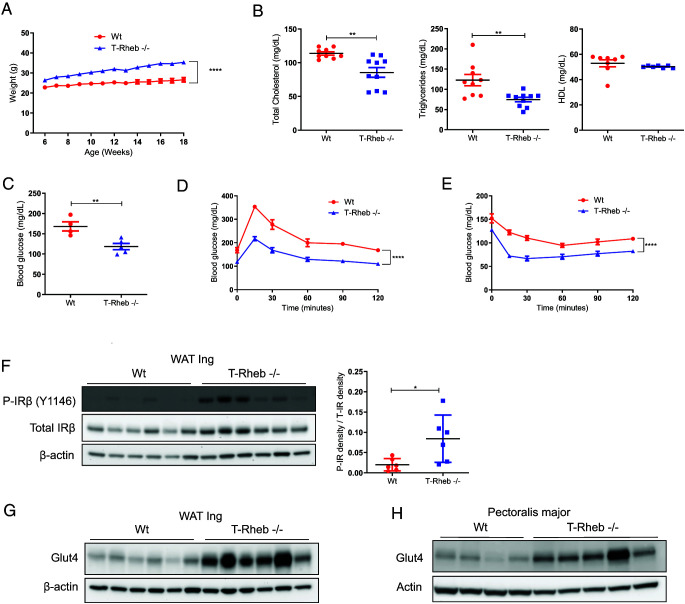
T-Rheb^−/−^ mice are heavier than control mice but demonstrate improved glucose and insulin tolerance. (**A**) Summary data of mouse weight on a normal chow diet starting at 6 wk of age. (**B**) Mice were fasted overnight; serum was isolated; and total cholesterol, triglycerides, and HDL were interrogated. (**C**) Mice were fasted for 7 h, and fasting blood glucose levels were obtained. (**D**) Mice were fasted for 7 h, then challenged with 1 g/kg glucose i.p., and blood glucose was measured over the indicated time course. (**E**) Mice were fasted for 2 h, then challenged with 0.5 U/kg insulin i.p., and blood glucose was measured over the indicated time course. (**F**) Left: Inguinal WAT (WAT Ing) was interrogated for phospho-IGF-I receptor β (P-IGF-I R β) (Y1131)/phospho-insulin receptor β (P-IR β) (Y1146) signaling via Western blot analysis. Right: The ratio of p-IR and total IR was determined by densitometry. (**G**) Western blot analysis performed on inguinal WAT from WT and T-Rheb^−/−^ mice. (**H**) Western blot analysis performed on pectoralis major skeletal muscle from WT and T-Rheb^−/−^ mice. Data are representative of three or more independent experiments of 5-mo-old mice (A, C–E) or 3-mo-old mice (B, F–H). An unpaired *t* test was performed for (B), (C), and (F), and repeated measures two-way ANOVA was performed for (A), (D), and (E) for statistical analysis (*p < 0.05, **p ≤ 0.01, ****p ≤ 0.0001). *n* = 4–10 mice per group. Data are mean ± SEM.

We have previously shown that T cells lacking the mTORC1 activator Rheb (T-Rheb^−/−^) fail to develop into CD4^+^ Th1 and Th17 as well as CD8^+^ effector cells (5, 7, 8). While breeding these mice, we noticed that T-Rheb^−/−^ mice were notably heavier than control mice over time (Fig. 1A). This observation of increased weight led us to characterize the overall metabolic phenotype of these mice. Interestingly, T-Rheb^−/−^ mice had significantly decreased total cholesterol and triglycerides, with similar HDL cholesterol, compared with WT mice (Fig. 1B). Thus, even though T-Rheb^−/−^ mice were heavier, they surprisingly displayed a healthier lipid profile.

With regard to glucose metabolism, initially on the basis of their increased weight, we hypothesized that these overweight mice might represent a model of type 2 diabetes. However, perhaps consistent with their healthier lipid profile, we found the exact opposite. Fasted T-Rheb^−/−^ mice had significantly lower fasting blood glucose than WT mice (Fig. 1C). Furthermore, T-Rheb^−/−^ mice showed improved glucose tolerance and insulin sensitivity (Fig. 1D, 1E), with no difference in serum levels of insulin or adiponectin (Supplemental Fig. 1A, 1B). They did have increased serum leptin (Supplemental Fig. 1C). Next, T-Rheb^−/−^ mice were tested to determine if they could maintain their enhanced glucose use when challenged with an HFD. Surprisingly, when T-Rheb^−/−^ mice were fed an HFD, they actually gained less weight than the control mice (Supplemental Fig. 1D). When HFD-fed mice were fasted, the T-Rheb^−/−^ mice had significantly lower fasting blood glucose than control animals (Supplemental Fig. 1E). Furthermore, HFD-fed T-Rheb^−/−^ mice had significantly improved glucose tolerance and insulin sensitivity (Supplemental Fig. 1F), as seen in the experiment with mice receiving a normal diet (Fig. 1D, 1E). Thus, T-Rheb^−/−^ mice did retain improved glucose homeostasis when challenged with the obesogenic diet. Overall, despite their obesity, T-Rheb^−/−^ mice did not exhibit a type 2 diabetes profile, but rather showed improved glucose tolerance and insulin sensitivity (even when challenged with an HFD).

WAT and skeletal muscle are major insulin-sensitive tissues in the body. Upon insulin stimulation, the insulin receptor triggers the translocation of Glut4 to the plasma membrane to facilitate glucose uptake into the tissues and lower the blood glucose (36). Strikingly, T-Rheb^−/−^ mice had significantly increased basal phosphorylation of the insulin receptor in inguinal WAT without any additional ex vivo stimulation (Fig. 1F). In addition, inguinal WAT and pectoralis major skeletal muscle of T-Rheb^−/−^ mice had greatly increased Glut4 protein levels (Fig. 1G, 1H). Collectively, these data indicate that heavier T-Rheb^−/−^ mice have the capacity to increase their tissue glucose uptake with enhanced insulin receptor activation and increased Glut4 protein.

### T-Rheb^−/−^ mice have increased total and beige adipose tissue

**FIGURE 2.**
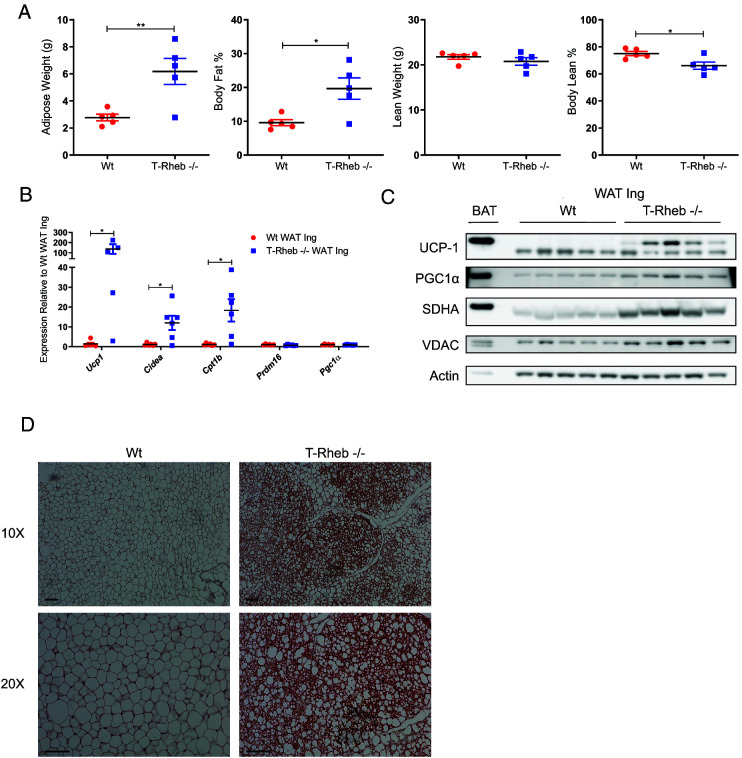
T-Rheb^−/−^ mice have increased adipose tissue and enhanced beige adipose tissue generation. (**A**) Total adipose weight, body fat percentage, lean weight, and body lean percentage were calculated via echo magnetic resonance imaging on 5-mo-old mice. (**B**) qRT-PCR was performed on inguinal WAT (WAT Ing) from WT and T-Rheb^−/−^ mice for the indicated genes. (**C**) Western blot analysis was performed on inguinal WAT (20 μg) and BAT (6 μg) to assess levels of the indicated proteins in WT and T-Rheb^−/−^ mice. (**D**) Inguinal WAT was stained for UCP-1 (brown) and counterstained with hematoxylin (blue) in both WT and T-Rheb^−/−^ mice. Scale bar, 10 µm. Data are representative of three or more independent experiments. An unpaired *t* test was performed for (A) and (B) (*p ≤ 0.05, **p ≤ 0.01). *n* = 4–6 mice/samples per group. Data are mean ± SEM.

Next, we tried to address the question why the T-Rheb^−/−^ mice appeared to be heavier. The most abundant and primary type of adipose tissue is WAT (37). WAT is crucial for energy storage and regulates systemic metabolism by producing adipokines (37, 38). A second distinct category of adipose tissue is brown adipose tissue (BAT) (37). BAT is enriched in mitochondria and expresses high levels of UCP-1, which generates heat via nonshivering thermogenesis (37). Beige adipose tissue is a third type of adipose tissue that is dispersed within WAT pads (37, 39, 40). Beige adipose tissue has characteristics of BAT, including increased mitochondria and UCP-1. There are numerous reports that posit a beneficial role of beige adipose tissue to restore energy balance in the face of caloric or energy surplus (37, 41). Because the T-Rheb^−/−^ mice are overweight (Fig. 1A), we decided to characterize their adipose tissue. T-Rheb^−/−^ mice had increased adipose tissue mass, resulting in increased body fat percentage, but with no difference in actual lean weight and a slight decrease in lean body mass percentage (Fig. 2A).

Because T-Rheb^−/−^ mice had increased adipose tissue (Fig. 2A), yet with an improved metabolic state (Fig. 1), their beige adipose tissue content was assessed. The search for beige adipose tissue focused on the inguinal WAT pad because this is a common site of beige adipose tissue development in mice (39). Indeed, T-Rheb^−/−^ mice had increased mRNA expression of beige adipose tissue markers, including *Ucp1*, *Cidea*, and *Cpt1b*, in the inguinal WAT fat pad; mRNA expression of *Prdm16* and *Pgc1*α was not altered (Fig. 2B). The increased *Ucp1* mRNA expression translated to increased UCP-1 protein levels as assessed by both Western blotting and immunohistochemistry (Fig. 2C, 2D). Despite no increase in *Pgc1*α mRNA expression, adipose tissue from T-Rheb^−/−^ mice increased protein expression of PGC1α, the master transcriptional coactivator of mitochondrial biogenesis, as well as the mitochondrial proteins SDHA and VDAC (Fig. 2C). Inguinal WAT of T-Rheb^−/−^ mice also exhibited a multilocular morphology indicative of beige adipose tissue (Fig. 2D). These data reveal that T-Rheb^−/−^ mice have more of the metabolically active beige adipose tissue.

### T-Rheb^−/−^ mice maintain an anti-inflammatory adipose tissue microenvironment

**FIGURE 3.**
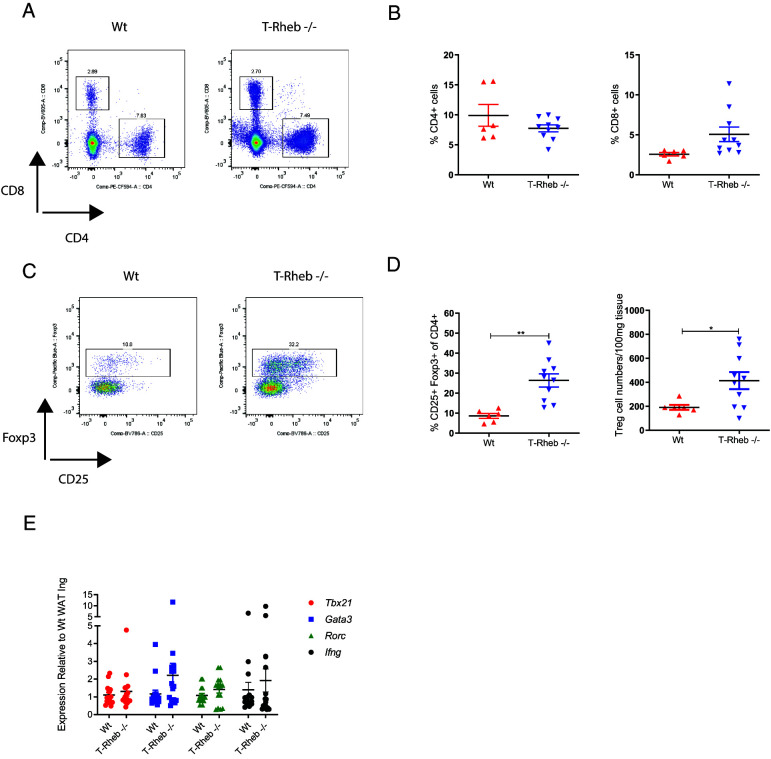
T-Rheb^−/−^ mice have increased adipose tissue infiltrating T regulatory cells. Cells were isolated from the perigonadal WAT (WAT Pg) of normal chow diet–fed WT or T-Rheb^−/−^ mice, and flow cytometric analysis was performed. All cells were gated on live-dead stain^−^, CD45^+^, and a single-cell gate. (**A**) Representative flow plots for CD4^+^ versus CD8^+^ cells. (**B**) Summary analysis of the percentage of CD4^+^ and CD8^+^ cells. (**C**) Representative flow plots for Foxp3^+^, CD25^+^ pregated on CD4^+^ (T regulatory cells). (**D**) Summary analysis of the percentage of CD4^+^, Foxp3^+^, CD25^+^ T regulatory cells, and numbers of T regulatory cells normalized per 100 mg of adipose tissue. (**E**) qRT-PCR was performed on inguinal WAT (WAT Ing) from WT and T-Rheb^−/−^ mice for the indicated genes. Data are three pooled experiments (E) or representative of three or more independent experiments (A–D). An unpaired *t* test was performed for statistical analysis (*p ≤ 0.05, **p ≤ 0.01). *n* = 6–10 mice per group (A–D) and 16 mice pooled per group (E). Data are mean ± SEM.

Adipose-infiltrating immune cells perform a critical role in the regulation of adipose tissue homeostasis (37, 42–44). In obesity, as adipose tissue mass increases, the normally anti-inflammatory immune microenvironment shifts toward a proinflammatory milieu (37, 42). The interplay between the immune system and obese-state adipose tissue is a potential mechanism for the development of insulin resistance, a key factor in type 2 diabetes (43). Therefore, we characterized the infiltrating T cells in WAT. T-Rheb^−/−^ and WT mice showed no significant differences in their percentages of infiltrating CD4^+^ or CD8^+^ T cells in perigonadal WAT (Fig. 3A, 3B). However, perigonadal WAT of T-Rheb^−/−^ mice had increased regulatory T cell (Foxp3^+^) infiltration (Fig. 3C, 3D). Regulatory T cells support an anti-inflammatory environment in adipose tissue that is beneficial in maintaining insulin sensitivity (37, 42). Additionally, we profiled the adipose tissue infiltrating CD4^+^ T cells to determine if there was any difference in Th cell subtypes between WT and T-Rheb^−/−^ mice. We observed no difference in *Tbx21*, *Gata3*, *Rorc*, or *Ifng* gene expression (Fig. 3E).

Macrophages are a major infiltrating cell type present by total number and percentage in adipose tissue. However, we did not find any significant differences in total macrophages (live, CD45^+^, CD11b^+^ F4/80^+^) in the WAT of WT and T-Rheb^−/−^ mice (Supplemental Fig. 2A, 2B). Additionally, when we evaluated adipose tissue infiltrating M1 (classically activated live, CD45^+^, CD11b^+^, F4/80^+^, CD11c^+^, CD206^−^) versus M2 (alternatively activated live, CD45^+^, CD11b^+^, F4/80^+^, CD11c^−^, CD206^+^) macrophages, we observed no significant differences (Supplemental Fig. 2C, 2D) (45). The immune microenvironment of WAT in T-Rheb^−/−^ mice is consistent with their improved insulin sensitivity and glucose homeostasis.

### KLK1b22 is expressed in Rheb^−/−^ T cells and enhances blood glucose clearance

**FIGURE 4.**
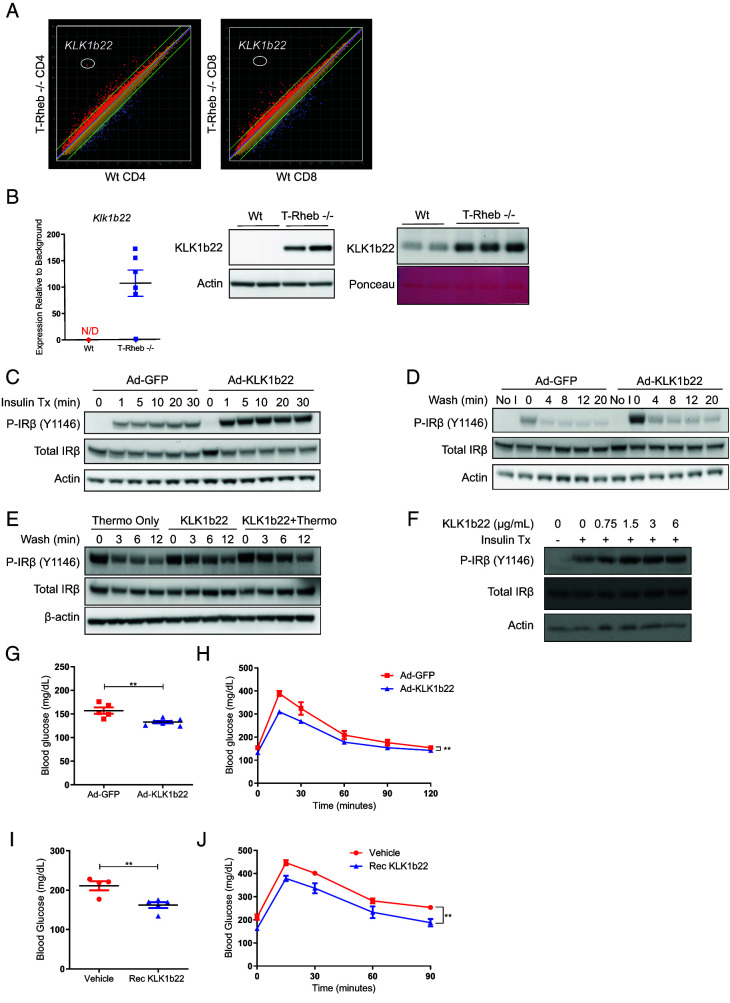
Rheb^−/−^ T cells express KLK1b22, which can decrease blood glucose and improve glucose tolerance. (**A**) Microarray analysis was performed on CD4^+^ and CD8^+^ T cells. (**B**) Splenocytes were analyzed via qRT-PCR (left) and Western blotting for KLK1b22 (middle). N/D, not detected. Serum was analyzed via Western blotting and Ponceau stain was used for the loading control. (**C**) HEK293T cells were transduced with adenovirus (Ad) GFP or KLK1b22, serum starved, and stimulated with insulin. Cells were collected, and Western blotting was performed. (**D**) Ad-transduced HEK293T cells were stimulated with insulin for 10 min, insulin was washed out, and cells were rested in HBSS for the indicated duration, at which point cells were collected and Western blotting was performed. (**E**) HEK293T cells were serum starved; pretreated with thermolysin, KLK1b22, or thermolysin-treated KLK1b22 for 30 min; then stimulated with insulin for 10 min, insulin was washed out, and cells were rested in HBSS for the indicated duration. Cells were collected, and Western blotting was performed. (**F**) Inguinal WAT was isolated from 6-wk-old WT C57BL/6J mice and chopped into small pieces. Adipose tissue was incubated with thermolysin-activated KLK1b22 and then stimulated with insulin. (**G**) Two-month-old WT C57BL/6J mice were injected with Ad-GFP or KLK1b22 i.v., and 3 wk later blood glucose was measured after a 7-h fast. (**H**) Mice from (G) were fasted for 7 h, then challenged with 2 g/kg glucose i.p., and blood glucose was measured. (**I**) Two-month-old WT C57BL/6J mice were fasted for 7 h and treated with recombinant KLK1b22; 45 min later, mice had blood glucose measured. (**J**) Mice from (I) were challenged with 2 g/kg glucose i.p., and blood glucose was measured. Microarray data are from one experiment. Other data are representative of two (B right, F, I, J) or three (B left, B middle, C, D, E, G, H) independent experiments. An unpaired *t* test was performed for (F) and (H), and repeated measures two-way ANOVA was performed for (G) and (I) (**p ≤ 0.01). *n* = 1–7 mice/samples per group. Data are mean ± SEM.

Because the T cell populations in WAT of T-Rheb^−/−^ and WT mice were different due to the deletion of Rheb, we performed microarray analysis to investigate differences in gene expression of the CD4^+^ and CD8^+^ T cells from these mice. Expression of the serine protease *Klk1b22* was greatly increased in both CD4^+^ and CD8^+^ T cells isolated from Rheb^−/−^ mice (Fig. 4A). This increased expression was also confirmed at both the mRNA and protein levels in splenocytes (Fig. 4B). Furthermore, T-Rheb^−/−^ mice also had increased serum levels of KLK1b22 (Fig. 4B, right). Thus, increased T cell expression of KLK1b22 in T-Rheb^−/−^ mice coincides with increased secreted KLK1b22 in their serum. To determine if Rheb deletion controlled KLK1b22 expression, we treated WT C57BL/6J T cells with rapamycin and deleted Rheb in mouse embryonic fibroblasts (data not shown). Neither of these conditions increased KLK1b22 expression. These findings were a little perplexing in that they suggested that although KLK1b22 expression was markedly increased in the T cells from the T-Rheb^−/−^ mice, mTOR inhibition or even the direct deletion of Rheb itself was not necessary for promoting KLK1b22 expression (see below for more on this topic). To see if *KLK1b22* was limited to T cells or expressed in other immune cells, we sorted out T cells, B cells, neutrophils, and monocytes from T-Rheb^−/−^ and WT mice. We found that T cells had the most expression of *KLK1b22* (Supplemental Fig. 3A), whereas B cells had some expression with monocytes and neutrophils had minimal expression.

Because T-Rheb^−/−^ mice showed increased insulin receptor phosphorylation in WAT (Fig. 1F), experiments were undertaken to determine if KLK1b22 could modulate phosphorylation of the insulin receptor. HEK293T cells were transduced with an adenovirus expressing KLK1b22 and stimulated with insulin in a protocol adapted from Krishnan et al. (35). Insulin receptor phosphorylation was increased at each time point in the adenovirus KLK1b22–treated cells versus adenovirus GFP control cells (Fig. 4C). Next, adenovirus-transduced cells were stimulated with insulin, followed by a wash to remove the insulin and increased lengths of rest time in HBSS to monitor signal decay. The adenovirus KLK1b22 cells maintained higher levels of insulin receptor phosphorylation than control cells at all time points (Fig. 4D). In further insulin washout experiments, recombinant KLK1b22 that was fully activated with thermolysin also increased and prolonged insulin receptor phosphorylation over time (Fig. 4E). Next, we performed an adipose tissue explant experiment to determine if recombinant KLK1b22 could also mediate insulin receptor phosphorylation in mouse adipose tissue ex vivo. When adipose tissue was pretreated with thermolysin-activated recombinant KLK1b22, we saw enhanced insulin receptor phosphorylation with insulin treatment compared with insulin treatment alone (Fig. 4F). These in vitro data (Fig. 4C–4E) are consistent with our in vivo adipose tissue data from T-Rheb^−/−^ mice (Fig. 1F), illustrating a role for KLK1b22 in mediating insulin receptor phosphorylation.

On the basis of these results that KLK1b22 could enhance and prolong insulin receptor phosphorylation in vitro and ex vivo (Fig. 4C–4F), we decided to investigate if KLK1b22 could affect blood glucose levels in vivo. WT C57BL/6J mice were administered injections with either adenovirus GFP or adenovirus KLK1b22 via the tail vein to systemically increase KLK1b22 levels. We observed the highest KLK1b22 expression in the livers of the Ad-KLK1b22 mice (Supplemental Fig. 3B). Two weeks later, mice were fasted, and we observed that the adenovirus KLK1b22 mice had fasting blood glucose levels that were significantly lower than those in adenovirus GFP control animals (Fig. 4G), an outcome similar to that seen in T-Rheb^−/−^ mice (Fig. 1C). Furthermore, adenovirus KLK1b22–injected mice challenged with a glucose bolus showed better glucose tolerance than control animals (Fig. 4H). Similarly, direct i.v. administration of recombinant KLK1b22 protein decreased fasting blood glucose versus vehicle control (Fig. 4I) and also improved glucose tolerance (Fig. 4J). These results indicate that KLK1b22 can modulate insulin receptor phosphorylation and improve glucose clearance in mice. Of note, no significant differences in adipose tissue amount or beige adipose tissue development were observed between the adenovirus GFP– and adenovirus KLK1b22–infected mice (data not shown). Consistent with the in vitro and ex vivo data (Fig. 4C–4F), these in vivo findings suggest that the improved glucose tolerance in T-Rheb^−/−^ mice is due at least in part to a direct action of KLK1b22.

### T-Rheb^−/−^, 129S1/SvImJ, and C3H/HeJ mouse T cells express KLK1b22 independent of Rheb deletion, which leads to improved glucose use

**FIGURE 5.**
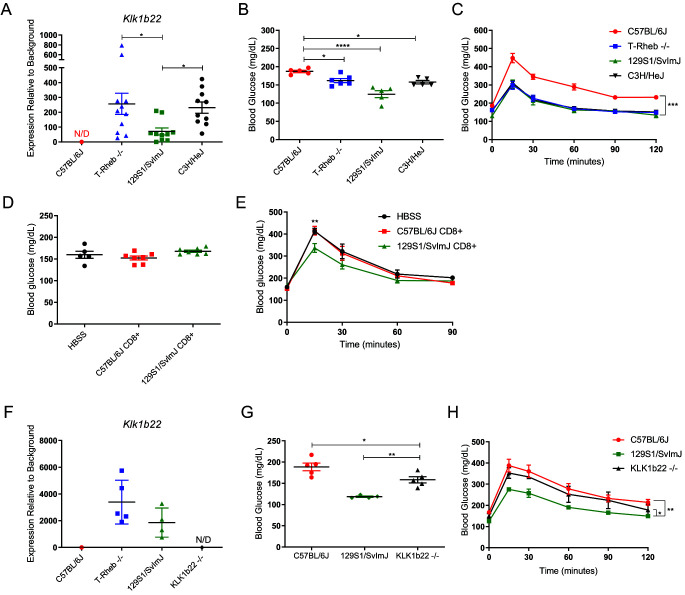
Rheb-independent KLK1b22 is a mediator of blood glucose and glucose tolerance. (**A**) qRT-PCR analysis for KLK1b22 was performed on lymphocytes from mouse blood in the indicated strains, N/D, not detected. (**B**) Two-month-old mice were fasted for 7 h, and fasting blood glucose levels were obtained. (**C**) Two-month-old mice were fasted for 7 h, then challenged with 2 g/kg glucose i.p., and blood glucose was measured at the indicated time points. (**D** and **E**) CD8^+^ T cells or HBSS was transferred into 3-mo-old Rag 2^−/−^ mice, and mice were interrogated 11 d after cell transfer. (D) Mice were fasted for 7 h, and fasting blood glucose was measured. (E) Mice were fasted for 7 h, then challenged with 2 g/kg glucose i.p., and blood glucose was measured at the indicated time points. (**F**) qRT-PCR analysis for KLK1b22 was performed on lymphocytes from mouse blood in the indicated strains or KLK1b22 KO mice (on a 129 background). N/D, not detected. (**G**) Two-month-old mice were fasted for 7 h, and fasting blood glucose levels were obtained. (**H**) Two-month-old mice were fasted for 7 h, then challenged with 2 g/kg glucose i.p., and blood glucose was measured at the indicated time points. Data are representative of two (D and E) or three (A–C, F–H) independent experiments. A Kruskal-Wallis test with Dunn’s multiple comparisons test was performed for (A), one-way ANOVA with Tukey’s multiple comparisons test was performed for (B) and (G), two-way repeated measures ANOVA was performed for (C) and (H), and two-way repeated measures ANOVA with Sidak’s multiple comparisons test was performed for (E) (*p ≤ 0.05, **p ≤ 0.01, ***p ≤ 0.001, ****p ≤ 0.0001). *n* = 3–11 mice/samples per group. Data are mean ± SEM.

Although it initially appeared that T cell–produced KLK1b22 was a phenomenon specific to the T-Rheb^−/−^ model, as mentioned above, we were perplexed that we could not recapitulate KLK1b22 expression by depleting Rheb. From searching the literature, we became aware of a study performed for the Immunological Genome Project characterizing 39 inbred mouse strains. Interestingly, and to our knowledge unnoticed by others, the Mouse Phenome Database reported increased *Klk1b22* expression in CD4^+^ T cells in two strains; *Klk1b22* expression was detected in CD4^+^ T cells of 129S1/SvImJ and C3H/HeJ mice (46). This gene was one of several examples of differences within the screened mouse cohort, and no significance or role was postulated for these differences. We confirmed *Klk1b22* expression in blood from WT 129S1/SvImJ, WT C3H/HeJ, and T-Rheb^−/−^ mice, whereas it was undetected in blood from WT C57BL/6J mice (Fig. 5A). Independently, a study by Champy and colleagues comparing C57BL/6J, 129SvPas, C3HeB/FeJ, and BALB/cByJ mice found improved glucose tolerance after an oral glucose challenge in the 129SvPas, C3HeB/FeJ, and BALB/cByJ mice (47). However, of note, in this study, there was no mechanism accounting for this observation and certainly no mention of KLK1b22. In our study, we found that 129S1/SvImJ, C3H/HeJ, and T-Rheb^−/−^ mice that produce KLK1b22 in their T cells had decreased fasting blood glucose compared with WT C57BL/6 mice (Fig. 5B). These three types of mice also had significantly better glucose tolerance than C57BL/6 mice in an i.p. glucose challenge (Fig. 5C). Notably, in relation to our initial observations, 129S1/SvImJ and C3H/HeJ mice expressed normal levels of Rheb in their T cells (data not shown). These data further support our initial observations that Rheb deletion is not the mechanism of KLK1b22 expression in the T-Rheb^−/−^ T cells (see more on this topic in the *Discussion* section).

Regardless of the exact role (or lack thereof) of Rheb, our data show that mice with T cells expressing high levels of KLK1b22 have improved fasting blood glucose and glucose tolerance (Fig. 1C, 1D, and Fig. 5B, 5C). In order to show a T cell–specific effect for KLK1b22, a T cell adoptive transfer system was used. T cells have previously been shown to play a beneficial role in the regulation of blood glucose in a *Rag1*^−/−^ model that lacks mature T and B cells (48). We took advantage of the high levels of expression of *Klk1b22* in T cells from 129S1/SvImJ mice (Fig. 5A) and transferred either CD8^+^ T cells from 129S1/SvImJ mice or C57BL/6J mice into recipient *Rag2*^−/−^ mice. Eleven days after T cell transfer, recipient *Rag2*^−/−^ mice were fasted, but no difference in fasting blood glucose was observed (Fig. 5D). However, when the mice were challenged with a glucose bolus, the *Rag2*^−/−^ mice that received KLK1b22^+^ CD8^+^ T cells (from 129S1/SvImJ mice) showed improved glucose tolerance (Fig. 5E). These observations support the hypothesis that T cells expressing KLK1b22 can improve systemic glucose tolerance.

Because C3H/HeJ and 129S1/SvImJ mice express KLK1b22 in their T cells, we investigated if these mice also had increased beige adipose tissue generation as was observed in the T-Rheb^−/−^ mice (Fig. 2B–2D). Curiously, T-Rheb^−/−^ and 129S1/SvImJ mice had increased expression of beige adipose tissue markers in their inguinal WAT, but C3H/HeJ mice did not (Supplemental Fig. 3C). Furthermore, the T-Rheb^−/−^ and 129S1/SvImJ mice had increased expression of UCP-1, PGC1α, and SDHA in their WAT, indicative of mitochondrial enrichment (Supplemental Fig. 3C–3E). T-Rheb^−/−^ and 129 mice showed histologic evidence of beige adipose tissue, indicated by multilocular morphology and UCP-1 expression (Supplemental Fig. 3E).

To further define the role of KLK1b22, a CRISPR-mediated KO (KLK1b22^−/−^) mouse was generated on the 129S1/SvlmJ background (Fig. 5F and Supplemental Fig. 4A). These KLK1b22^−/−^ mice had increased fasting blood glucose as compared with WT 129S1/SvImJ mice (Fig. 5G). Furthermore, these mice also had significantly decreased glucose tolerance at a level similar to WT C57BL/6J mice (Fig. 5H). These data demonstrate a role for KLK1b22 to increase glucose tolerance in mice. However, when we interrogated beige adipose tissue markers, the KLK1b22^−/−^ mice had *Ucp1*, *Cidea*, and *Cpt1b* expression similar to that of WT 129S1/SvImJ mice (Supplemental Fig. 4B). Furthermore, the KLK1b22 KO mice had UCP-1 protein levels similar to those of the WT 129S1/SvImJ mice (Supplemental Fig. 4C, 4D). Thus, in concert with the outcomes from the adenovirus experiments to increase KLK1b22 (Fig. 4), these KLK1b22 KO studies indicate a role for KLK1b22 to enhance glucose clearance, but it is not responsible for the beige adipose tissue generation. These results suggest that KLK1b22 can regulate systemic glucose use and that KLK1b22-producing T cells can improve glucose tolerance.

## Discussion

In this study, we identify striking systemic metabolic changes in T-Rheb^−/−^ mice. These include decreased fasting glucose, increased glucose tolerance and insulin sensitivity on a normal chow diet and an HFD, and markedly increased beige fat generation. The T cells from these mice ectopically express KLK1b22, a member of the KLK family that is normally found at high levels in the salivary gland. Interestingly, the deletion of Rheb has no role in modulating KLK1b22 expression.

Our data support a role for KLK1b22 in promoting glucose tolerance. Indeed, overexpression of KLK1b22 or the addition of activated KLK1b22 protein increased insulin receptor activation and kinetics in vitro and ex vivo (Fig. 4C–4F). These data are also consistent with the increased insulin receptor activation we observed in the adipose tissue of the T-Rheb^−/−^ mice (Fig. 1F). Furthermore, overexpression of KLK1b22 in WT mice with adenovirus led to increased glucose tolerance (Fig. 4G, 4H). Thus, our studies reveal (to our knowledge) a novel potent role for KLK1b22 in regulating glucose metabolism. Although this KLK is mouse specific, these findings provide the potential to exploit these observations for the development of a novel drug to treat type 2 diabetes. Indeed, the drug exenatide, which is used for type 2 diabetes, is a synthetic version of exendin-4, which was originally found in Gila monster venom and mimics human GLP-1 (49).

The Mouse Phenome Database reported that 129S1/SvImJ and C3H/HeJ T cells express *Klk1b22* (46). On the basis of our own data, we hypothesized that these mouse strains would demonstrate increased glucose tolerance. Indeed, this is exactly what we observed (Fig. 5A–5C), and, furthermore, such an observation of increased glucose tolerance was previously described (independent of any association with KLK1b22 or T cells) by Champy and colleagues (47). Notably, we could not replicate KLK1b22 expression by simply inhibiting mTORC1 (e.g., with rapamycin or in T-Raptor^−/−^ mice) or by simply deleting Rheb. Thus, we do not think that this ectopic expression is related to mTORC1 signaling (data not shown). We now believe that our identification of T cell–derived KLK1b22 as a regulator of systemic glucose tolerance is most likely a serendipitous artifact of using mice on the 129 background. Nonetheless, we took advantage of these findings by using CRISPR to create 129S1/SvImJ KLK1b22^−/−^ mice. Genetic deletion of *Klk1b22* abrogated the increased glucose tolerance in these mice (Fig. 5F–5H). Thus, we propose that KLK1b22 produced and secreted by T cells mediates improved glucose tolerance in mice. Furthermore, we demonstrate that transfer of KLK1b22-expressing T cells can improve glucose tolerance (Fig. 5E). These T cells can infiltrate insulin-sensitive tissues, such as adipose tissue (Fig. 3), and could act in a paracrine manner by secreting KLK1b22 locally to mediate the improvement in glucose use. There could be a systemic role for KLK1b22 as well, because we did observe increased KLK1b22 in the serum of T-Rheb^−/−^ supportive of such a role (Fig. 4B). Importantly, we observed no difference in serum insulin levels in T-Rheb^−/−^ mice (Supplemental Fig. 1A); thus, the increase in KLK1b22 in our T-Rheb^−/−^ model does not modulate total serum insulin levels.

Previous data on KLKs has been contradictory with regard to their effect on blood glucose (12, 15–17, 50–53). In type 1 diabetes models, virus-mediated expression of KLK1 or recombinant KLK1 administration has been shown to decrease blood glucose and delay onset of the disease (15, 16). In preliminary studies, our laboratory has found that T-Rheb^−/−^ mice can defend against the development of type 1 diabetes (data not shown). Kolodka et al. have shown that treatment of mice and rats with recombinant KLK1 lowers blood glucose (17). Likewise, we show that recombinant KLK1b22 and Ad-KLK1b22 can lower blood glucose (Fig. 4). Conversely, KLK7 can cleave insulin, impairing glucose tolerance (51). This effect can be blocked by the adipokine visceral adipose tissue–derived serpin (vaspin) (50, 51). However, in our study, KLK1b22 expression did not affect the serum insulin levels in T-Rheb^−/−^ mice (Supplemental Fig. 1A). Zieger and colleagues have recently shown that adipose tissue–specific KO of KLK7 leads to decreased adipose tissue inflammation, decreased weight, and improved glucose tolerance when mice are fed an HFD (52). This KO model contrasts with our present study, which used increased KLK1b22 expression. The study by Zieger et al. illustrates a detrimental effect of adipose tissue–produced KLK7 on blood glucose homeostasis, whereas we report a beneficial effect of KLK1b22 (Figs. 4, 5). The authors propose that inhibitors of KLK7 could be beneficial for metabolic disease (52). Furthermore, Potier and colleagues have shown that KLK1^−/−^ mice (tissue KLK^−/−^) had no effect on blood glucose (53). In contrast, our KLK1b22^−/−^ data show increased fasting blood glucose and decreased glucose tolerance (Fig 5G, 5H). Of note, their KLK1^−/−^ mice are on a 129/Sv-C57/BL6 background, whereas our KLK1b22^−/−^ mouse is on a 129S1/SvImJ background. It is important to note that C57BL/6J mice do not express KLK1b22 in their T cells, whereas 129S1/SvImJ mice do (Fig. 5A) (46, 53).

Adipose tissue is a crucial organ for the maintenance of glucose homeostasis, and the immune system undertakes a fundamental role in its regulation (37, 42). T-Rheb^−/−^ mice on a normal diet have increased adipose tissue but are paradoxically healthier, as illustrated by decreased fasting blood glucose, triglycerides, and total cholesterol (Fig. 1). Many pharmacological agents, transcription factors, hormones, and other stressors that decrease fat and body weight gain have been demonstrated to induce beige adipose tissue (39, 54–56). Additionally, BAT has high *Ucp-1* expression and has been shown to uptake triglycerides from the blood (57, 58). The decreased serum triglycerides in our T-Rheb^−/−^ mice may be consistent with this mechanism. Adipose tissue is an insulin-sensitive tissue, and insulin signaling increases cellular surface localization of Glut4 (36). T-Rheb^−/−^ mice demonstrate an increased phospho-insulin signal in inguinal WAT and increased Glut4 expression (Fig. 1F, 1G), which provides a mechanistic explanation for the decreased blood glucose (Fig. 1C). Yet, importantly, whereas genetic deletion of KLK1b22 led to a decrease in glucose tolerance (Fig. 5F–5H), this was in the setting of maintained beige adipose tissue (Supplemental Fig. 4B–4D). Thus, although the abundance of brown/beige fat in the 129S1/SvImJ and T-Rheb^−/−^ certainly can contribute to their ability to maintain weight and handle glucose, our data suggest that KLK1b22 promotes glucose tolerance independent of increased brown/beige fat generation.

Stolarczyk et al. report a T-bet^−/−^ mouse model that has increased adipose tissue weight, improved glucose and insulin tolerance, and increased serum leptin, similar to our T-Rheb^−/−^ model (59). Because T-bet (like Rheb) plays an integral role in the generation of Th1 and CD8^+^ effector cells, this is an intriguing parallel finding. However, these T-bet^−/−^ mice have decreased total CD45^+^, CD4^+^, and CD8^+^ infiltrating immune cells in the perigonadal WAT but increased T regulatory cells in this compartment, whereas our findings show no difference in CD4^+^ and CD8^+^ T cells but increased T regulatory cells in T-Rheb^−/−^perigonadal WAT (Fig. 3). T regulatory cells are known to be beneficial in promoting insulin sensitivity by promoting an anti-inflammatory adipose tissue microenvironment (37, 42, 43). However, the mechanism for the increase in such T cells and whether these cells are actually the cause of increased beige adipose tissue are unclear to us. To this end, although we believe our observations strongly support a role for KLK1b22 in regulating glucose tolerance, we have yet to determine the precise mechanism whereby the T-Rheb^−/−^ and 129S1/SvImJ mice have increased beige adipose tissue, whereas the C3H/HeJ mice do not. Whether this can be explained by an additional factor, by an immunologic mechanism, or by a combination of both remains to be determined. Further studies involving the target of KLK1b22 and pursuit of the mechanisms involved in beige adipose tissue generation not only will provide important insight into the regulation of metabolism but also will have important implications for the development of novel compounds to treat diabetes and obesity.

## Supplementary Material

Supplemental Figures 1 (PDF)
