# Supplementary material for: Serendipitous Discovery of T Cell–Produced KLK1b22 as a Regulator of Systemic Metabolism
Source: Immunohorizons. 2023 Jun 26;7(6):493–507. doi: 10.4049/immunohorizons.2300016 (PMC10580127; doi:10.4049/immunohorizons.2300016)
Supplement: Supplemental Figures 1 (PDF) [file IH_2300016_Supplemental_1.pdf]

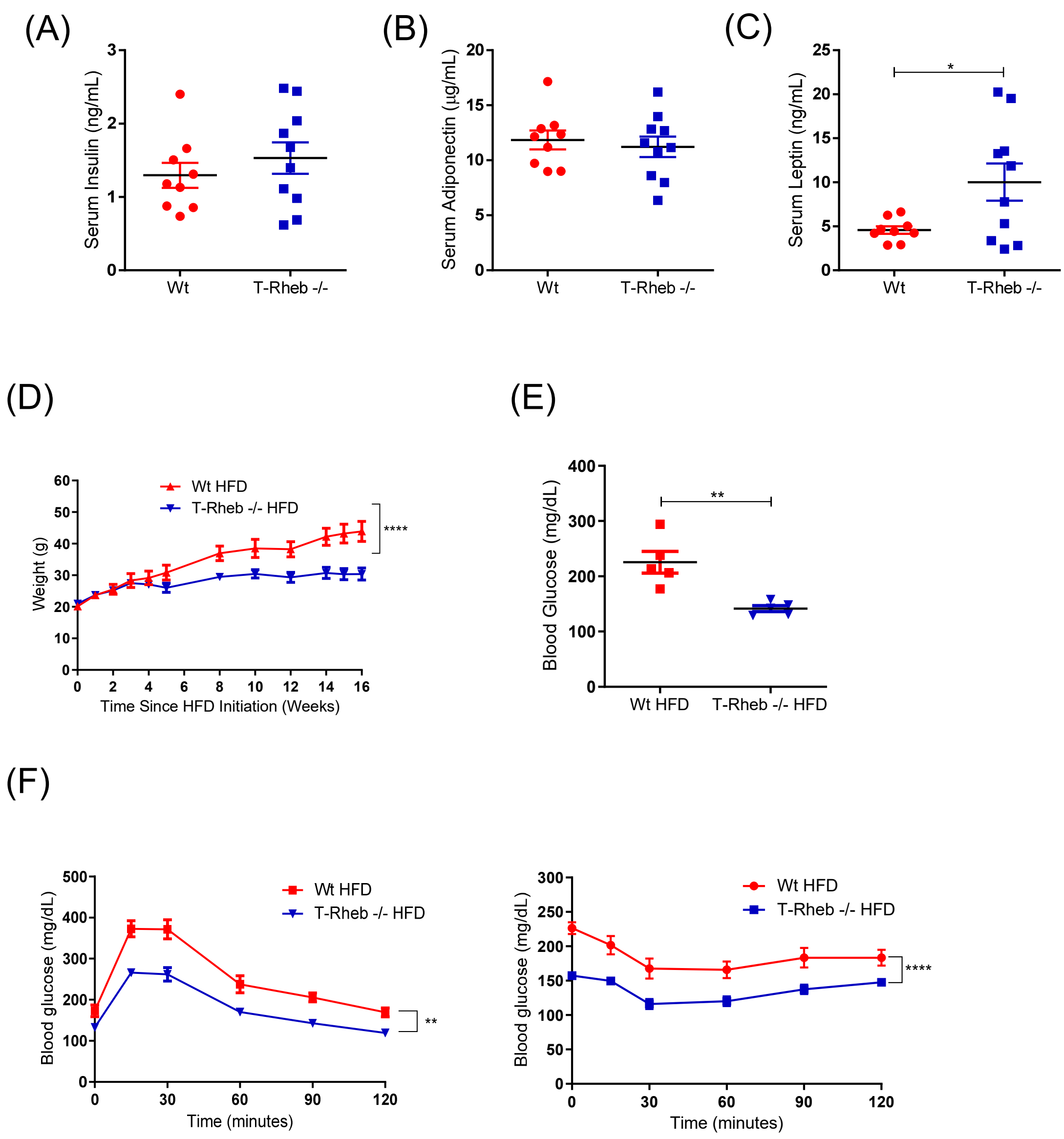

Supplemental Figure 1. Three-month-old mice were fasted overnight and serum isolated from the blood. (A) Insulin, (B) adiponectin, and (C) leptin concentrations were determined via ELISA analysis of the serum. (D-F) Six-week-old mice were fed high fat diet (HFD) for 3 months prior to the following studies. (D) Mice were weighed starting at 6 weeks of age. (E) Mice were fasted for 7 hours and fasting blood glucose levels were obtained. (F) Left - mice were fasted for 7 hours, challenged with 1g/kg glucose IP, and blood glucose levels were measured. Right - mice were fasted for 2 hours, challenged with 1U/kg insulin IP, and blood glucose measured. Data are representative of 2 independent experiments (D-F) or 3 independent experiments (A-C). An unpaired t-test was performed for statistical analysis for C and E. A repeated measures two-way ANOVA was performed for D and F. (* $P \leq 0.05$, ** $P \leq 0.01$, **** $P \leq 0.0001$). N = 5-10 mice per group. Data are mean +/- SEM.

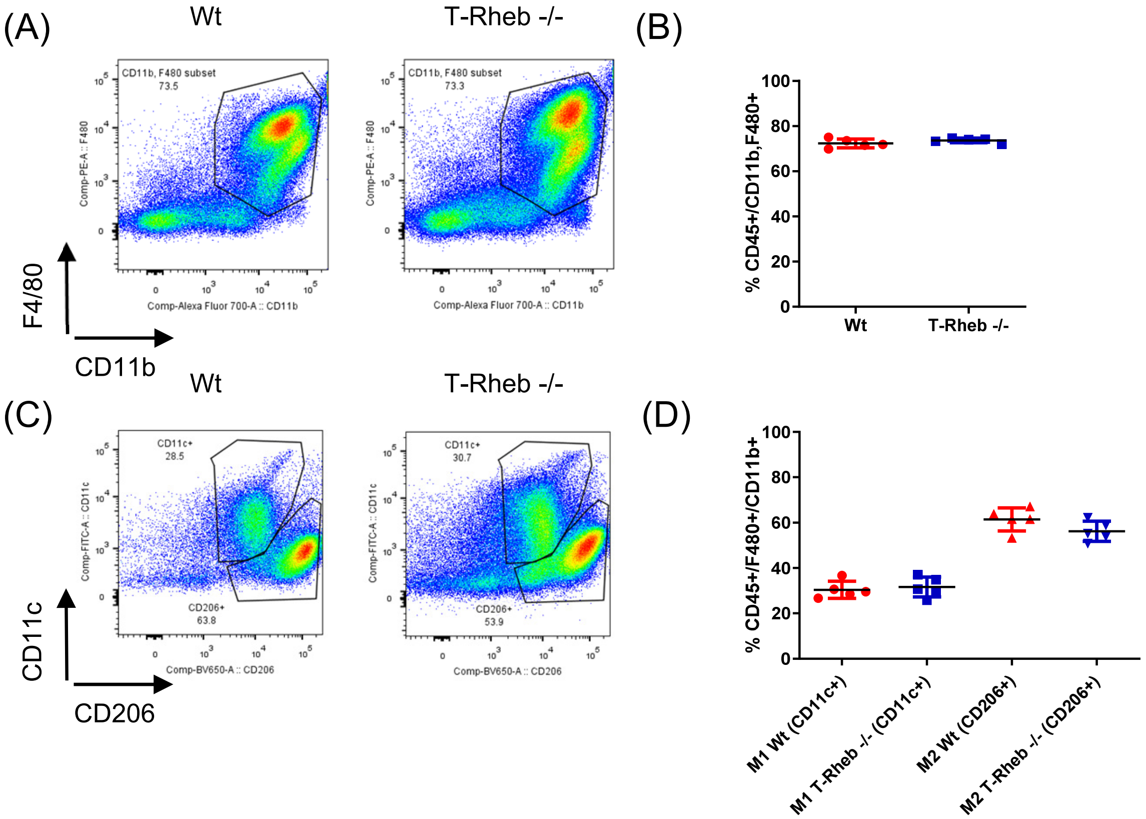

Supplemental Figure 2. Cells were isolated from the perigonadal WAT (WAT Pg) of normal chow diet-fed Wt or T-Rheb -/- mice and flow cytometric analysis was performed. All cells were gated on live-dead stain, CD45+, and a single cell gate. (A) Representative flow plots for F4/80 and CD11b double positive cells. (B) Summary analysis of percentage of F4/80 and CD11b double positive cells. (C) Representative flow plots for CD11c+ (M1) versus CD206+ (M2) cells pre-gated on CD45, CD11b, and F4/80. (D) Summary analysis of percentage of CD11c+ (M1) versus CD206+ (M2) cells pre-gated on CD45, CD11b, and F4/80. Data are representative of 3 or more independent experiments (A-D). N = 5 mice per group. Data are mean +/- SEM.

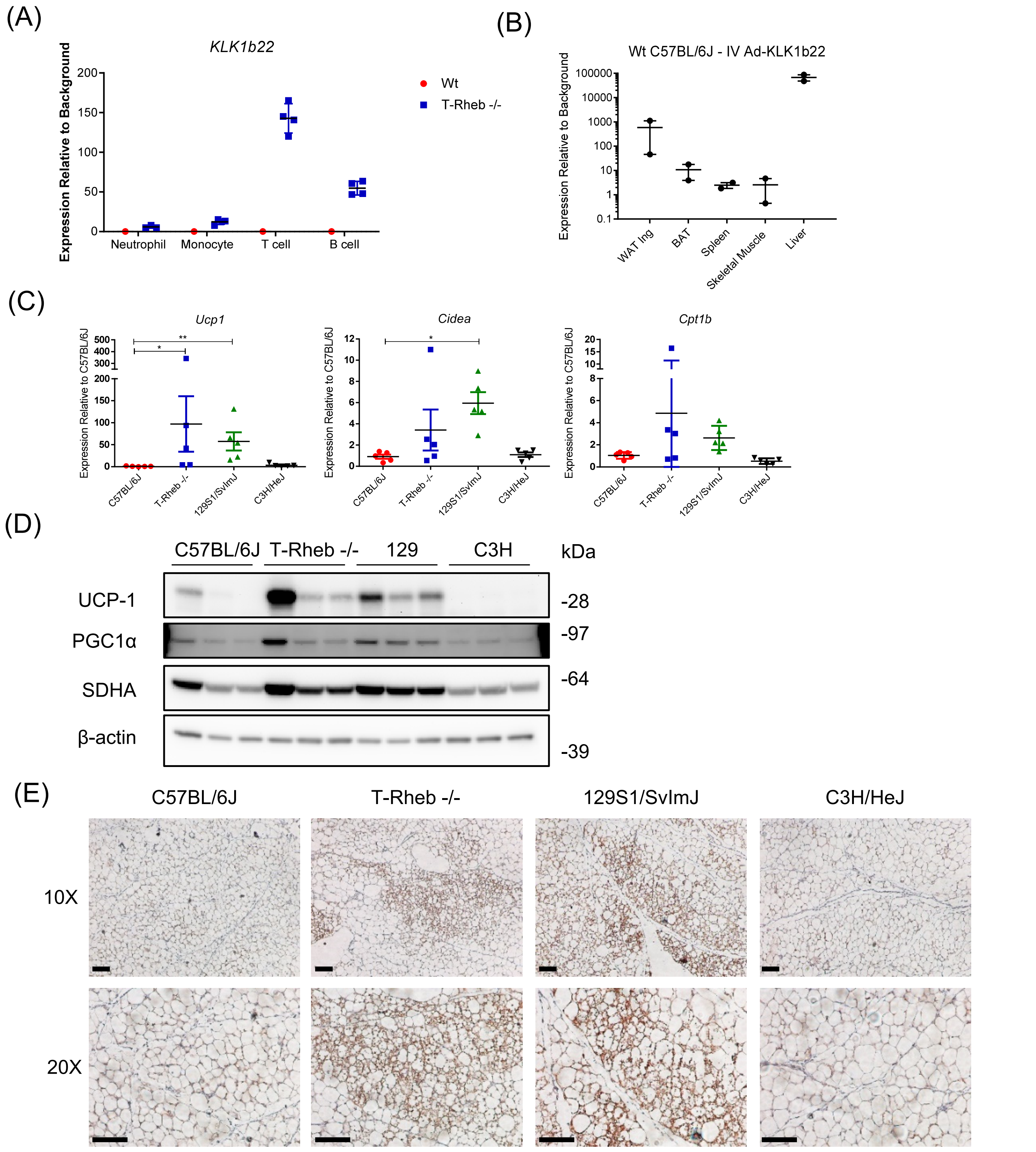

Supplemental Figure 3. (A) Cells were sorted from spleen (T cells (CD3+) and B cells (CD19+)) and bone marrow (neutrophils (CD11b+, Ly6G+) and monocytes (CD11b+, Ly6C+)) and qRT-PCR analysis performed for *KLK1b22* expression. (B) Wt C57B6/J mice were injected IV with Ad-KLK1b22. After 2 weeks the indicated tissues were harvested, RNA isolated, and qRT-PCR performed for *KLK1b22*. Inguinal white adipose tissue (WAT Ing). Brown adipose tissue (BAT). (C) Inguinal WAT was isolated and qRT-PCR analysis was performed for beige adipose tissue genes. (D) Inguinal WAT was isolated and Western Blot analysis was performed for beige adipose tissue genes. (E) Inguinal WAT was stained for UCP-1 (brown) and counterstained with hematoxylin (blue). Scale bar = 10μm. Data are representative of 2 independent (A) or 3 independent experiments (B-D), or 1 experiment with samples from 5 mice (E). A Kruskal-Wallis test with Dunn's multiple comparisons test was performed for A (* $P \leq 0.05$, ** $P \leq 0.01$). N = 1-5 mice/samples per group. Data are mean +/- SEM.

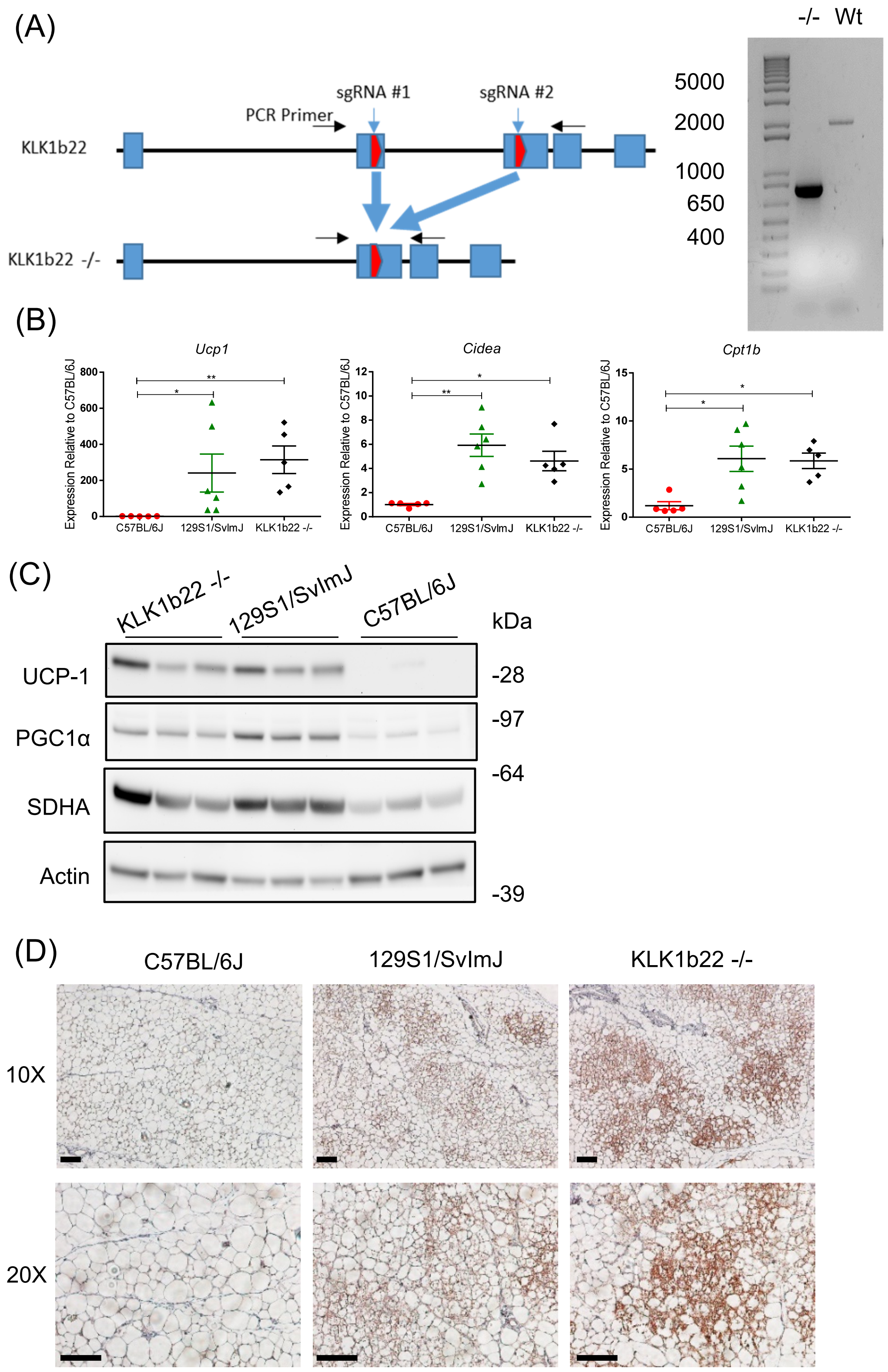

Supplemental Figure 4. (A) Schematic for KLK1b22 gene sgRNA targets (exon 2 and exon 3) (Left). PCR amplification was performed with primers flanking the total region of exon 2 and 3, gel was run to show CRISPR KO obtained (right). (B) Inguinal WAT was isolated and qRT-PCR analysis was performed for beige adipose tissue genes. (C) Inguinal WAT was isolated and Western Blot analysis was performed for beige adipose tissue genes. (D) Inguinal WAT was stained for UCP-1 (brown) and counterstained with hematoxylin (blue). Scale bar = 10μm. Data are representative of 2 experiments (A-C) or 1 experiment with samples from 5 mice (D). A one-way ANOVA with a Tukey's multiple comparisons test was performed for B (*Cidea* and *Cpt1b*), and a Kruskal-Wallis test with Dunn's multiple comparisons test was performed for B (*Ucp-1*). (* $P \leq 0.05$, ** $P \leq 0.01$). N = 3-6 mice/samples per group. Data are mean +/- SEM.
